# Universal molecular structures in natural dissolved organic matter

**DOI:** 10.1038/s41467-018-05665-9

**Published:** 2018-08-09

**Authors:** Maren Zark, Thorsten Dittmar

**Affiliations:** 1 Research Group for Marine Geochemistry (ICBM-MPI Bridging Group), Carl von Ossietzky University of Oldenburg, Institute for Chemistry and Biology of the Marine Environment (ICBM), Carl-von-Ossietzky-Str. 9-11, 26129 Oldenburg, Germany; 2 Helmholtz Institute for Functional Marine Biodiversity at the University of Oldenburg (HIFMB), Ammerländer Heerstraße 231, 26129 Oldenburg, Germany

## Abstract

Natural dissolved organic matter (DOM) comprises a broad range of dissolved organic molecules in aquatic systems and is among the most complex molecular mixtures known. Here we show, by comparing detailed structural fingerprints of individual molecular formulae in DOM from a set of four marine and one freshwater environments, that a major component of DOM is molecularly indistinguishable in these diverse samples. Molecular conformity was not only apparent by the co-occurrence of thousands of identical molecular formulae, but also by identical structural features of those isomers that collectively represent a molecular formula. The presence of a large pool of compounds with identical structural features in DOM is likely the result of a cascade of degradation processes or common synthetic pathways that ultimately lead to the formation of a universal background, regardless of origin and history of the organic material. This novel insight impacts our understanding of long-term turnover of DOM as the underlying mechanisms are possibly universal.

## Abstract (web summary)

Dissolved organic matter (DOM) in aquatic systems is among the most complex molecular mixtures known. Here the authors show that a major component in DOM is molecularly indistinguishable in marine and freshwater environments, which could reflect universal mechanisms behind long-term DOM turnover.

## Introduction

DOM comprises a complex molecular mixture^1–3^ being largely the remnant of microbial and abiotic reworking of organic matter originally derived from terrestrial or aquatic primary producers^4^. Mostly because of this molecular complexity, full structural elucidation of DOM has been impossible to date. The structures of only a few percent of the many hundreds of thousands of the individual compounds in DOM are known^5^. The remarkable advance of ultrahigh-resolution analytical techniques, namely Fourier-transform ion cyclotron resonance mass spectrometry (FT-ICR-MS) and high-field nuclear magnetic resonance spectroscopy (NMR), has allowed fundamentally novel insights into the molecular composition of DOM. More than 10,000 molecular formulae and hundreds of structural features have been identified with their help to date^1,6^. The molecular structures behind these molecular formulae, and the spatial arrangement of the identified structural features, however, are still largely unknown and remain an ongoing analytical challenge^5,7^.

The original source exhibits a strong influence on DOM molecular composition^8^ and, consequently, on its susceptibility to microbial and abiotic transformations^9^. For instance, DOM in freshwater is often enriched in lignin-derived polyphenols that originate from vascular plant debris^10,11^. The abundance of these polyphenols renders the waters brown in color as typical for many rivers, lakes or wetlands. Marine DOM is comparably poor in these compounds^10,12^. Aside from the obvious source-specific molecular properties of DOM, on which a rich body of literature exists, there is a component in DOM that is molecularly indistinguishable across all natural aquatic systems^1,8,13–16^. Evidence for this universal component in DOM was found with help of ultrahigh-resolution FT-ICR-MS and multidimensional nuclear magnetic resonance (NMR). Thousands of molecular formulae found by FT-ICR-MS in DOM in the deep ocean are also present in rivers, lakes, wetlands and even degraded plant leachate and microbial cultures^8,13,17,18^. A common structural feature, as indicated by a combination of NMR and FT-ICR-MS, are carboxylic-rich alicyclic moieties (CRAM)^15^ and material derived from linear terpenoids (MDLT)^16^. It was hypothesized that long-term degradation processes such as a cascade of photo-degradation together with microbial mediated transformation of DOM leads source-independently to these common structural features^8,13,19,20^. This molecularly indistinguishable fraction of DOM may withstand decomposition over millennia and remain in solution during transport from the site of production at the sea surface or on the continents into the deep sea^18,21^. However, a largely unexplored level of molecular diversity exists for DOM because each molecular formula is represented by a range of different isomers, i.e., compounds with the same molecular formula but different molecular structures^5,22,23^. Similarly, there is a high degree of freedom to arrange the structural features identified in DOM via NMR. Possibly, the molecular similarity of the universal DOM background may simply be the reflection of our inability to fully characterize DOM on a structural level. This lack of knowledge is of concern because it prevents a truly mechanistic understanding of organic matter cycling in freshwater and marine systems.

Here we took the analytical challenge to assess the molecular diversity of DOM beyond the molecular formula level by performing extensive experiments on individual molecular formulae in an ultrahigh-resolution FT-ICR-MS. This was done to test the hypothesis that DOM in the various aquatic environments and from the various sources only apparently shares identical molecular features, and that differences emerge on an isomeric level. The results demonstrate the opposite and identical structural features are identified for the isomers that represent a molecular formula across all tested environments. This indicates that the underling mechanism of DOM turnover is universal for this component of DOM.

## Results

### Analysis of DOM from different aquatic environments

To compare DOM from most different aquatic environments on a structural molecular level we performed conventional and fragmentation FT-ICR-MS analyses on DOM that was isolated via solid-phase extraction from different waters in the ocean and on land. These samples are representative in their molecular composition for a wide range of aquatic and marine systems (Supplementary Fig. 1). Samples with similar mass spectra, but different structures, can be clearly distinguished by this approach. For example, DOM produced by marine microbial communities in culture experiments is very similar to DOM in the ocean regarding molecular formulae^17,24^, but fragmentation experiments uncovered different molecular structures behind the same molecular formulae in such experiments^25^. Exemplary analysis of benzenetricarboxylic acid isomers with identical molecular formulae as model compounds illustrated that even slight structural differences lead to sharply different fragmentation patterns in FT-ICR-MS (Supplementary Fig. 2).

**Fig. 1:**
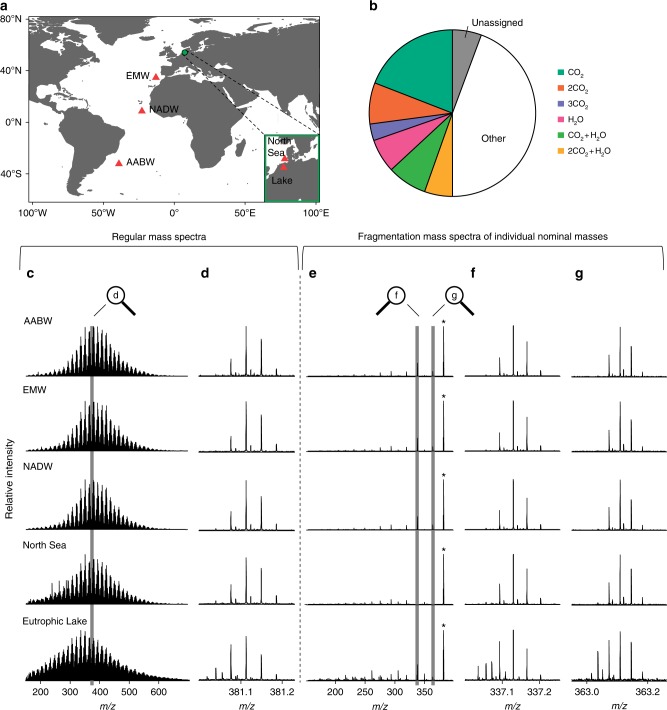
FT-ICR mass spectra of DOM samples, broad band scans and fragmentation experiments. **a** Map of sampling sites^53^. **b** Most abundant fragment ions occurring in dissolved organic matter (DOM) fragmentation experiments as percentage of total intensity (named according to the corresponding neutral loss). **c** Full range mass spectra showing bell-shape intensity distribution of detected masses. **d** Detected ions with nominal mass *m*/*z* 381 zoomed in from full range mass spectra, exemplary for the six fragmented nominal masses. **e** Full fragmentation patterns of *m*/*z* 381, exemplarily. **f** Fragment ions resulting from neutral loss of CO_2_ and **g** from neutral loss of H_2_O, zoomed in from the respective mass range in full fragmentation pattern. Precursor ions are indicated by asterisks (*)

Out of the 6166 molecular formulae that were detected in the deep sea, the coastal ocean, and a peat lake (Fig. 1a) 2531 formulae (41%) co-occurred in all samples (Fig. 1c). The percentage of this fraction of common molecular formulae relative to the total number of formulae in a given sample was highest for Eurafrican Mediterranean Water (87%) and lowest for the eutrophic peat lake (47%, Supplementary Table 1).

We chose six individual nominal masses (*m*/*z* 365, 379, 367, 381, 369, 383) for fragmentation experiments from full range mass spectra of the five DOM samples (Fig. 1a, c). These nominal masses represented a total of 217 molecular formulae of which 54 co-occurred in all the five samples. Molecular formulae were isolated from these full range mass spectra within a window of one nominal mass (Fig. 1d) and fragmented via collision and detected in the FT-ICR-MS. All these formulae lost mainly carbon dioxide (CO_2_) from decarboxylation and water (H_2_O) as a result of alcohol dehydration or formation of anhydrides^26–30^ (Fig. 1b, e–g and Supplementary Fig. 3). Carboxyl and hydroxyl groups were thus the predominant functional groups of DOM in all our samples. We searched for a total of 324 different CO_2_ and H_2_O fragments of which 185 were detected. Other neutral losses were carbon monoxide (CO) and methanol (CH_3_OH). In addition, very minor fragmentation signals in terrestrially influenced samples likely originated from polyphenolic compounds, a compositional feature indicative of the presence of vascular plant debris. Despite a clear terrigenous origin, fragmentation of functional groups that are typical for lignin (methoxy groups)^31^ were not observed. This is likely because these compounds are readily degraded^32,33^ and were already removed on their journey from soils to the lake or into the coastal ocean. The qualitative evaluation of fragmentation patterns, i.e., the type of occurring neutral losses, agrees with earlier fragmentation studies on DOM^26–30^.

### In-depth structural analysis of common DOM compounds

**Fig. 2:**
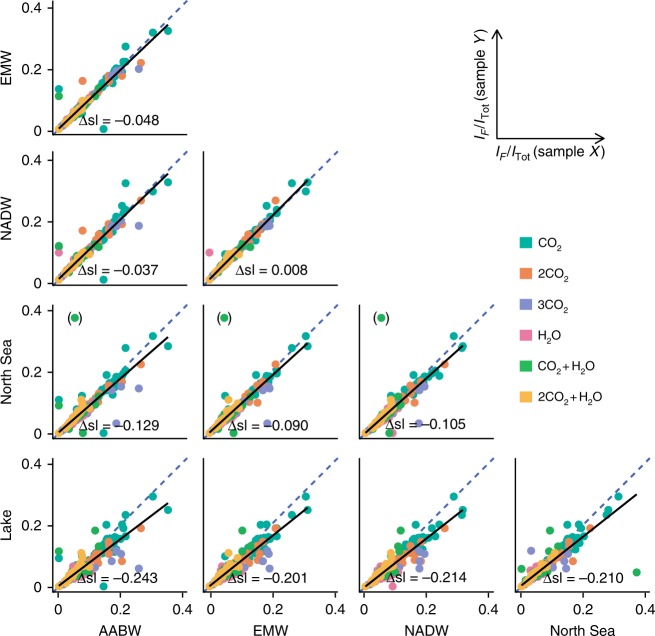
Structural relation of DOM from different environments. Linear regression analysis of relative fragment ion intensities from common molecular formulae shows the structural variance between two samples indicated by the significance of the correlation (all *p* < 0.0001, *n* = 324) and the deviation of the slope (Δsl) from ideal conformity (blue dashed lines). One outlier (in parentheses) was excluded from regression analysis. AABW Antarctic Bottom Water; EMW Eurafrican Mediterranean Water; NADW North Atlantic Deep Water

Detailed structural information on DOM compounds is crucial to better predict its potential impacts on the global climate system^34,35^. While the overall fragment mass patterns give a good overview on the type of potential functionalities, differences in relative fragment ion intensities are indicative of even subtle structural differences as it was shown for the exemplary set of isomers (Supplementary Fig. 2). We hence extended the analysis towards an essential next step, the quantitative evaluation of fragment ion intensities, which provides a data basis for detailed statistical evaluation. A linear regression analysis was performed to examine how closely the intensities of common fragments matched in any two samples. Therefore, relative signal intensities were calculated for all assigned fragment ions that occurred from common masses. It must be noted that only the main fragment ions were considered, that account for around 60% of the total fragment intensity. Relative fragment intensity (*I*_F_/*I*_Tot_) is here defined as the FT-ICR-MS signal intensity of the fragment ion (*I*_F_) divided by the sum intensity of all major signals (*I*_Tot_), which includes the precursor ion and fragments attributed to that precursor. In a comparison of relative fragment ion intensities, a slope of 1 is expected if two samples contain identical compounds. We found highly significant positive correlations (*r* > 0.91, *p* < 0.0001, *n* = 324) between the fragment intensities of all samples and only very minor and non-significant deviations from a slope of one, with Δsl ranging from 0.008 (NADW/EMW) to up to 0.243 (Lake/AABW) (Fig. 2).

**Fig. 3:**
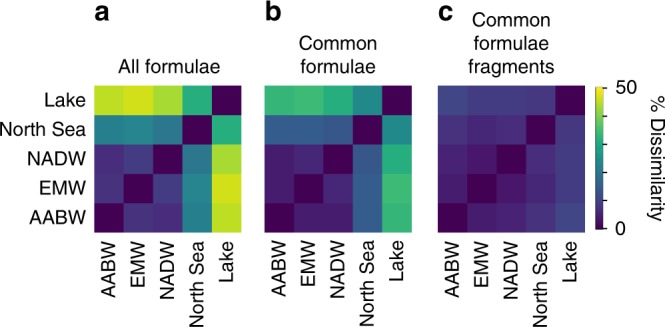
Molecular dissimilarity of DOM from different environments on different compositional levels. Bray–Curtis dissimilarity based on relative FT-ICR-MS signal intensities of **a** all detected, unfragmented molecular formulae in full range mass spectra (*n* = 6166) and **b** common molecular formulae in full range mass spectra (*n* = 2531). **c** Bray–Curtis dissimilarity based on relative FT-ICR-MS signal intensities of fragments (*n* = 324) of the most intense common molecular formulae (*n* = 54) that were selected for fragmentation experiments. AABW Antarctic Bottom Water, NADW North Atlantic Deep Water, EMW Eurafrican Mediterranean Water

We further performed statistical dissimilarity analyses (Bray–Curtis) based on FT-ICR-MS signal intensities to describe the molecular differences between the DOM samples on the molecular formula level and on the structural level via fragmentation. The analysis was performed first for all detected, unfragmented molecular formulae (6166) in the full-range, unfragmented mass spectra (Fig. 3a). The highest dissimilarity on this level was observed between Eurafrican Mediterranean Water and the eutrophic peat lake sample (47%). The abundance of typical terrigenous polyphenols in the peat lake is one reason for this high level of molecular dissimilarity. The dissimilarity analysis of common molecular formulae that were present in all samples also showed a high degree of dissimilarity between the samples (up to 34%, Fig. 3b). This is because the molecular formulae occurred in different abundance pattern in the samples, e.g., more unsaturated compounds were more abundant in the lake than in the deep sea (see also Supplementary Fig. 4). The fragment ions that are resulting from common molecular formulae, however, were highly similar among the samples, and the Bray–Curtis dissimilarity never exceeded 11% (Fig. 3c) which is within the range of our analytical variability (8% for replicate analyses of the same DOM sample). In other words, identical molecular formulae not only lost the same molecular fragments, but these fragments occurred at the same abundances in all samples.

The high similarity of relative fragment ion intensities is remarkable, considering the different origins of the samples. Regardless of origin, compounds that shared the same molecular formula in vastly different aquatic environments also shared indistinguishable structural features, even when assessed by the most advanced analytical techniques. This similarity did not only apply to the type of functional groups, but most likely also to their position in the given molecule. Our experiments with model compounds (Supplementary Fig. 2) demonstrated that even a minor difference in structure leads to vastly different fragmentation patterns in the FT-ICR-MS.

### Molecular diversity

**Fig. 4:**
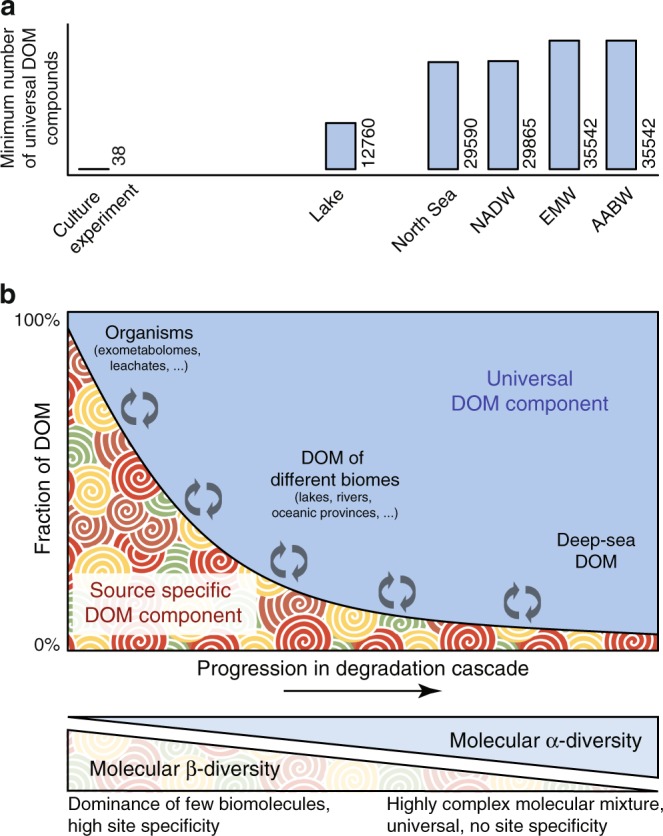
Conceptual view of DOM composition along the degradation and mixing cascade. **a** Minimum number of universal DOM compounds that are detectable in the respective samples. The number of compounds was estimated by multiplying the minimum number of isomers by the number of detected molecular formulae common to the samples with highest degradation state, AABW and EMW (see also Supplementary Fig. 5). **b** A molecularly universal DOM background is present already at early stages of degradation, even in unconnected aquatic environments. A source-specific component leads to a high molecular β-diversity among these environments. In the course of degradation and mixing, the source-specific component is preferentially removed and after thousands of years of degradation in the deep sea, the universal background emerges as a predominant feature. This background is molecularly highly diverse, in terms of richness or number of different compounds (high α-diversity), but universal across aquatic environments (low β-diversity)

We further explored our mass spectrometry data with a novel statistical approach that allows an estimate of the minimum number of isomers behind a given molecular formula^5^. The exact number of isomers remains unknown, but this lower limit estimate allows a comparison of isomeric diversity between samples. The approach is based on principals of the central limit theorem. For this, the number of carboxyl groups is estimated from molecular fragmentation patterns and, independently, from molecular formula information alone^5^. With increasing number of isomers, the match between the two independent estimates increases because of intrinsic averaging on a molecular formula level. The level of isomeric diversity, as indicated by the correlation coefficient between the two estimates, increased from the lake sample to deep water masses and the Eurafrican Mediterranean Water (Fig. 4a, Supplementary Fig. 5a–e).

## Discussion

DOM produced by diverse microbial communities in culture experiments^25^, in comparison, showed far less diversity of isomers than natural DOM (Fig. 4a, Supplementary Fig. 5f). In other words, the number of isomers increased with degradation state of the DOM sample. This together with recent indications that single compounds in DOM occur in vanishingly low concentrations in the deep ocean^3,5^ supports the scenario of DOM diversification (in terms of molecular richness^36^) over degradation and mixing, and a decreasing occurrence of site-specific DOM compounds. It has recently been shown that time is a driver over DOM molecular composition and diversity across aquatic ecosystems^37^. In theory, it is also possible that the 54 fragmented formulae share a common set of source-material in the different environments, e.g., specific biomolecules from terrestrial organic matter input. However, we chose the compounds across the entire spectrum of DOM compounds and not from a subgroup with specific molecular composition (see Supplementary Fig. 4) and it is hence unlikely that all fragmented compounds share the same precursor. It is more likely that universal biosynthetic pathways exist across the range of tested environments that may ultimately lead to the formation of similar carbon skeletons with high degrees of carboxyl and hydroxyl substitutions. Further, the hypothesized diversification is in agreement with other concepts on DOM diversity. The iconic River Continuum Concept for example, states that DOM diversity decreases from the headwater streams to the river mouth with only the refractory compounds being the leftover after rapid degradation of labile compounds^38^. Creed et al.^39^ further developed a new model to explain the DOM variability observed in river systems and they hypothesize an increasing chemostasis of DOM, meaning a higher spatial and temporal heterogeneity, with increasing stream order. In analogy to this theoretical model, we interpret our molecular level information on DOM from different geographical origin.

We introduce two levels of diversity (Fig. 4b): First, the diversity between ecosystems, here termed molecular β-diversity in analogy to ecological concepts. This is the level of diversity that the River Continuum concept refers to and which is also resembled in the view of the river as a chemostat^39^. We acknowledge that the full level of molecular β-diversity was not assessed in this study, and future studies are encouraged to test the theoretical β-diversity concept on the full landscape level.

At the same time, the structural diversity on the isomer level increases through the stepwise formation of a complex mixture with a high number of isomers behind each molecular formula. This diversity (or richness) that is inherent of a specific ecosystem is termed molecular α-diversity. It is important to note that as in ecology, α-diversity was determined in our study by a random sample. The comparison of α-diversity between different samples is only possible if the size of the samples is the same, since the number of species (or molecules), and thus the diversity, usually increases with increasing sample size. We accounted for these conceptual considerations by analyzing all samples at the same DOC concentration and collected the same number of FT-ICR-MS scans for each sample.

Cascades of degradation processes and mixing in aquatic environments likely increase molecular α-diversity through the stepwise formation of a complex mixture with a high number of isomers behind each molecular formula^8,13,14^. Degradation processes could be abiotic (photo- or thermal degradation) or biotic, and particularly microbial reworking is an important process in this context. Marine microbes remove DOM from the water, but they also produce new DOM. In fact, the DOM released by marine microbes is more diverse than the initial substrates and, thus, they contribute to molecular diversification^17,25^. Part of the source-specific DOM component, however, seems to be resistant towards photo- and year-long biodegradation^40^, and long timescales are necessary for the observed prevalence of the universal DOM component in the deep sea. Previous studies were stating the same hypothesis, that common processes may form a large pool of similar DOM structures across different environments^8,39^. Our structural comparison of DOM from different marine and freshwater environments and our estimates of isomeric diversity are confirmative of these ideas presented previously^8,39^. The question arises whether the same holds true for N and S containing compounds. We did not exclude these compounds in our study, however, only few of the fragmented formulae contain these heteroelements as the original unfragmented signals are usually not as abundant as those with formulae containing only C, H, and O. We would like to acknowledge that even though the new data presented cannot conclusively settle the discussion on DOM molecular diversity, it represents a major step towards an improved understanding of the structure of the DOM pool.

Our study represents the first comparison of comprehensive DOM molecular structural features in different aquatic environments, including the deep sea and a eutrophic peat lake. Within our analytical window, we found a component in DOM that is molecularly indistinguishable across the globe. This novel insight impacts our understanding of long-term turnover of DOM as the underlying mechanisms are possibly universal, at least for this component. Affinity of consumers towards common biomolecules is one of the commonalities of degradation processes in all aquatic environments, but the structural similarity of DOM seems to go far beyond what can be explained by this process.

## Methods

### Sampling sites and sample preparation

Samples were collected from five different sites (Fig. 1a). Exact positions, volumes and water depths are displayed in Supplementary Table 1. Antarctic Bottom Water (AABW), North Atlantic Deep Water (NADW), and Eurafrican Mediterranean Water (EMW) were collected onboard the German research vessel Polarstern on cruise leg ANT XXVIII/5 in 2012 using a rosette sampler. The samples were pooled from two to three stations to obtain sufficient volume. AABW and NADW are deep-water masses with a refractory DOM signature^41^ but of different origin as NADW is formed in the North Atlantic and AABW in the Southern Ocean. Highly saline EMW from the outflow of the Mediterranean Sea is younger than oceanic deep-water masses but also characterized by a refractory DOM signal^42^. A sample from the North Sea, a continental shelf sea in Europe that contains a terrigenous component and freshly produced DOM^43^, was taken with a bucket from the sea surface. Water from a eutrophic peat lake was collected in January 2011 in Meyerhausen, Germany, and is presumably dominated by terrestrial vascular plant decomposition products^44^.

### Dissolved organic carbon analysis

Water was filtered through 0.7 µm GF/F glass microfiber filters (Whatman, pre-combusted 400°C, 4 h), acidified with HCl (25%, analysis grade, Carl Roth) to pH 2 and stored at 4°C in the dark. Analysis of DOC concentrations was performed via high-temperature catalytic oxidation method^45^ using a Shimadzu (Japan) TOC-VCPH/CPN Total Organic Carbon Analyzer equipped with ASI-V auto sampler. We controlled the accuracy of the measurement for every run by analyzing deep-sea reference samples provided by the University of Miami (Dennis Hansell). The error of DOC was below 2.8 µmol C L^-1^.

### DOM extraction and FT-ICR mass spectrometry

All samples (0.5–12 L, depending on the DOC concentration of the sample) were extracted via solid phase extraction using a commercially available PPL sorbent (Agilent)^46^. Procedural blanks were prepared by processing ultrapure water the same way as DOM samples. DOC concentrations in the resulting extracts were below the detection limit. We performed mass spectrometric analysis of DOM extracts via FT-ICR-MS on a solariX Fourier transform ion cyclotron resonance mass spectrometer with 15 Tesla magnet (Bruker Daltonics, USA). The system was equipped with an electrospray ionization source (ESI, Bruker Apollo II) applied in negative ionization mode. Methanol extracts were mixed with ultrapure water (50:50 v/v) for FT-ICR-MS analysis and diluted to a final DOC concentration of 20 mg C L^−1^. For each measurement we accumulated 500 scans in the mass window of 150–2000 Da. Including test runs and the analysis of NEqPIW deep-sea reference, we needed more than 600 h of instrument time to acquire all full-range, isolation and fragmentation mass spectra. We calibrated spectra internally with a reference mass list using Data Analysis software Version 4.0 SP4. The mass error of the calibration was <0.06 ppm for all samples. We used MatLab routines developed by our working group for molecular formula assignment and further data processing. Only peaks with a signal-to-noise ratio of S/N = 5 or higher that fulfilled the criteria by Koch et al.^47^ and Rossel et al.^13^ were considered including the elements C, H, O, N, S, and P with respect to the following criteria C_0–60_, H_0–∞_, N_0–4_, S_0–2_, P_0–1_, O/C_max_ = 1, and H/C_min_ = 0.3. All molecules were detected as singly charged ions. To test the reproducibility and stability of the FT-ICR-MS analysis, SPE-DOM from North Equatorial Pacific Intermediate Water (NEqPIW) was analyzed with the same settings twice a day^25,48^. Finally, we normalized peak intensities of the peaks with assigned molecular formula to the sum of peak intensities for multivariate statistical analysis. A total of 9794 resolved masses of singly charged, intact compounds were detected via FT-ICR-MS. Thereof, 6166 masses could be assigned to molecular formulae. Most of the remaining detected masses (96%) could be assigned to a molecular formula by including ^13^C_1_ and ^15^N_1_ into the list of considered elements and can therefore be related to isotopologues (analogs of the previously assigned molecular formula containing one ^13^C or ^15^N). Molecular categories according to criteria modified after Šantl-Temkiv et al.^49^ were calculated for all detected molecular formulae largely based on the modified aromaticity index AI_mod_^50^. The following groups were differentiated: combustion-derived polycyclic aromates (Black Carbon; AI_mod_ ≥ 0.666, no N, S, P), oxygen rich polyphenols (0.666 > AI_mod_ < 0.5, O/C > 0.5), oxygen poor polyphenols (0.666 > AI_mod_ < 0.5, O/C ≤ 0.5), highly unsaturated compounds (AI_mod_ < 0.5, H/C < 1.5, O/C < 0.9), and aliphatic compounds (2.0 ≥ H/C ≥ 1.5, O/C < 0.9, no N) (Supplementary Table 2). Consistent with the respective origin, the eutrophic peat lake sample showed the highest number of molecular formulae of polyphenols (e.g., lignin-derived compounds) and combustion-derived, polycondensed aromates (black carbon), while EMW showed the highest percentage of highly unsaturated compounds. Further, we were able to show that the samples are representative for a wide range from terrestrial to marine environments by performing principal component analyses including endmember samples from the deep-sea and from ten of the largest world rivers (see Supplementary Fig. 6)

### ESI FT-ICR tandem mass spectrometry experiments

For fragmentation, ions were first isolated in 0.4 Da windows in a quadrupole unit of the FT-ICR-MS. Ions were then transferred into a hexapole unit, where they accumulated for 2 s and collided with neutral argon gas. The molecular formulae that were chosen for fragmentation represent all the major molecular categories (Supplementary Table 2), and belong to different CH_2_ homologous series. Thus, they are representative for the molecular diversity of DOM. The mass range around 370 Da was chosen because the high signal intensities enable acquisition of the most detailed structural fingerprints. Fragmentation of DOM samples was carried out with methanol extracts that were mixed with ultrapure water (50:50 v/v) to give a final concentration of 100 mg C L^−1^. For each spectrum 150 scans were acquired with a data acquisition size of 4 Mword. The collision voltage was 15.0 V and the detection range was set from *m*/*z* 125–2000. Collision induced fragmentation in the quadrupole with subsequent detection via FT-ICR-MS resulted in a total of 1953 distinct molecular fragments, of which 265 derived from the 54 molecular formulae that were common in all samples.

As model compounds, three isomers of benzenetricarboxylic acid were analyzed at a concentration of 10 mg C L^−1^, with the mass window for isolation in the quadrupole set to 0.2 Da, 300 scans were acquired for each compound, and the collision voltage was set at 5.0 V. To calibrate fragmentation mass spectra, we subtracted the exact masses of known occurring neutral losses of water, carbon dioxide, and combinations thereof (*m*/*z* 18.01056, 43.98983, 62.00039, 87.97966, 105.99022, 131.96949) from the masses of the ions detected on the respective isolated nominal mass (Fig. 1d). The resulting exact masses of potentially occurring fragment ions were then used for internal calibration. With this method, formula assignment to the fragment ions and identification of the respective precursor ion became possible, based on the exact mass differences^25^. Formula assignment was performed the same way as described for the full range mass spectra.

### Statistical analysis of FT-ICR-MS data

Fragment ions with large signal intensities are more reproducible (smaller error in signal intensity). Therefore, we included only the most intense fragment ions for the statistical analysis, which were resulting from neutral losses of mainly CO_2_, H_2_O and multiples thereof. Other fragments include for example multiples of C_n_ compounds. For better comparison we calculated the relative peak intensity *I*_F_/*I*_Tot_ for each individual fragment ion. Individual peak intensities were divided by the sum of the intensities of all major fragment peaks (corresponding to the neutral losses of CO_2_, 2CO_2_, 3CO_2_, H_2_O, CO_2_+H_2_O, 2CO_2_+H_2_O, CH_3_OH, CO_2_+CH_3_OH, 2CO_2_+CH_3_OH) and the precursor ion peak intensity. Based on these relative peak intensities, a linear regression model was calculated (Fig. 2). For this analysis, only fragment ions were considered that were the result of fragmentation of common molecular formulae detected in the full range FT-ICR mass spectra across all samples. A common detection limit for all samples and individual precursor ions was defined as the minimum ratio of the signal of the respective precursor ion and analytical noise. To describe structural differences between the individual samples we used matrices of normalized signal intensities of all masses detected in full range mass spectra, masses with common molecular formulae, and the normalized relative fragment intensities from fragmentation experiments to compute dissimilarity matrices based on Bray–Curtis compositional dissimilarity (Fig. 3). Normalization was done by dividing the respective values by the sum of all peak intensities in the sample. For dissimilarity analysis of the common fragment intensities, we considered the same data as for the linear regression model. All statistical analyses were performed with the software package R (Version 3.0.2, package “vegan”)^51^.

### Data availability

DOC and sample metadata are included in this published article and its Supplementary Information files. Mass spectrometric data has been deposited in the PANGAEA Open Access library (10.1594/PANGAEA.888852)^52^.

## Electronic supplementary material

Supplementary Information
