# Supplementary material for: Universal molecular structures in natural dissolved organic matter
Source: Nat Commun. 2018 Aug 9;9:3178. doi: 10.1038/s41467-018-05665-9 (PMC6085399; doi:10.1038/s41467-018-05665-9)
Supplement: Supplementary file 1 — Supplementary Information [file 41467_2018_5665_MOESM1_ESM.pdf]

Supplementary information:

Universal molecular structures in natural dissolved organic matter

Maren Zark[1*], Thorsten Dittmar[1,2*]

[1] Research Group for Marine Geochemistry (ICBM-MPI Bridging Group), Carl von Ossietzky University of Oldenburg, Institute for Chemistry and Biology of the Marine Environment (ICBM), Carl-von-Ossietzky-Str. 9-11, D-26129 Oldenburg, Germany.

[2] Helmholtz Institute for Functional Marine Biodiversity at the University of Oldenburg (HIFMB), Ammerländer Heerstraße 231, D-26129 Oldenburg, Germany.

*Correspondence to: maren.zark@uni-oldenburg.de, thorsten.dittmar@uni-oldenburg.de

This .pdf file includes:

Supplementary Figures 1 - 6

Supplementary Tables 1 & 2

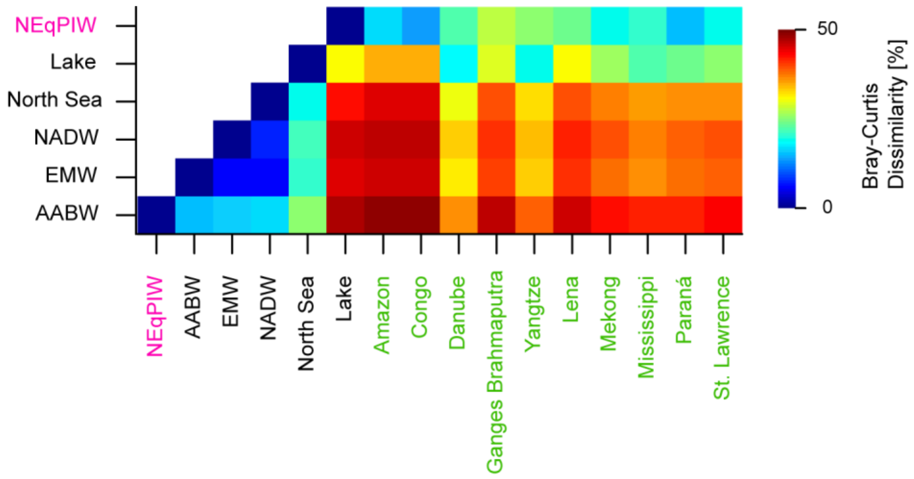

Supplementary Fig. 1: Classification of the set of DOM samples analyzed in this study within the range of DOM from marine to terrestrial environments based on molecular dissimilarity. The samples analyzed in this study were chosen from a range from marine to terrestrial environments. Bray-Curtis dissimilarity based on relative FT-ICR-MS signal intensities of all detected common molecular formulae in full range mass spectra (n=5,553) shows that North Sea represents a mixed sample and that the Lake water is highly similar in its molecular composition to 10 of the largest rivers worldwide. Data from the 10 rivers are from Riedel et al.[40] NEqPIW is North Equatorial Pacific Intermediate Water and is one of the oldest oceanic water masses worldwide.

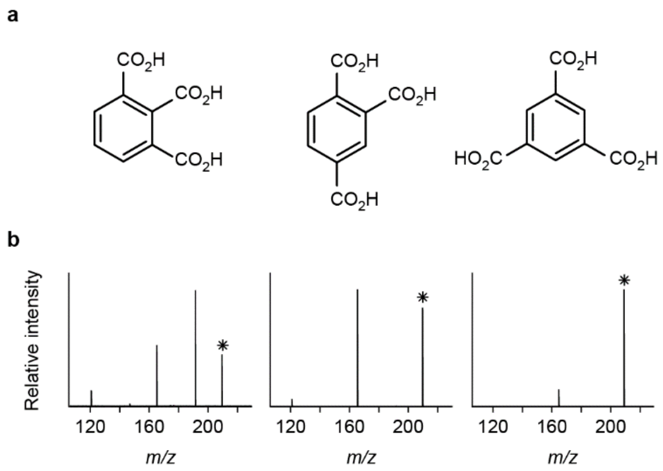

Supplementary Fig. 2. Fragmentation FT-ICR mass spectra of benzenetricarboxylic acid isomers with identical molecular formulae. (a) Chemical structures of the individual compounds. (b) Fragment ions (m/z 121, 165, 191) of the same precursor ion (m/z 209) show different signal intensities according to the position of the carboxylic groups attached to the benzene ring. Precursor ions are indicated by asterisks (*) in each spectrum. Despite the same fragmentation conditions applied for each analysis, the three main fragment ions occurred in very different intensities. Fragments are the result of multiple neutral losses of water and carbon dioxide at the mass to charge ratios *m/z* 191 (loss of 1 H_2O), 165 (loss of 1 CO_2) and 121 (loss of 2 CO_2). The results are highly reproducible for the given instrumental settings.

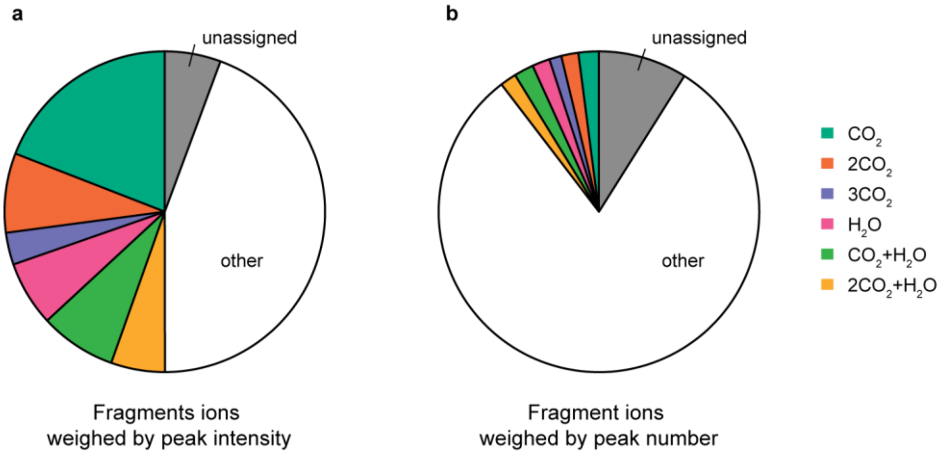

Supplementary Fig. 3: Most abundant neutral losses observed in fragmentation experiments of DOM samples as percentage of (a) total intensity, and (b) total peak number. Fragment ions that could not be unambiguously assigned to a molecular formula were labeled "unassigned" and fragment ions that were not related to the most abundant neutral losses (mainly CO_2 and H_2O) were not considered for statistical evaluation and thus labeled "other". This is because there are many small peaks that contribute only little to the overall signal intensity and which have inherently larger errors of signal intensities compared to larger peaks.

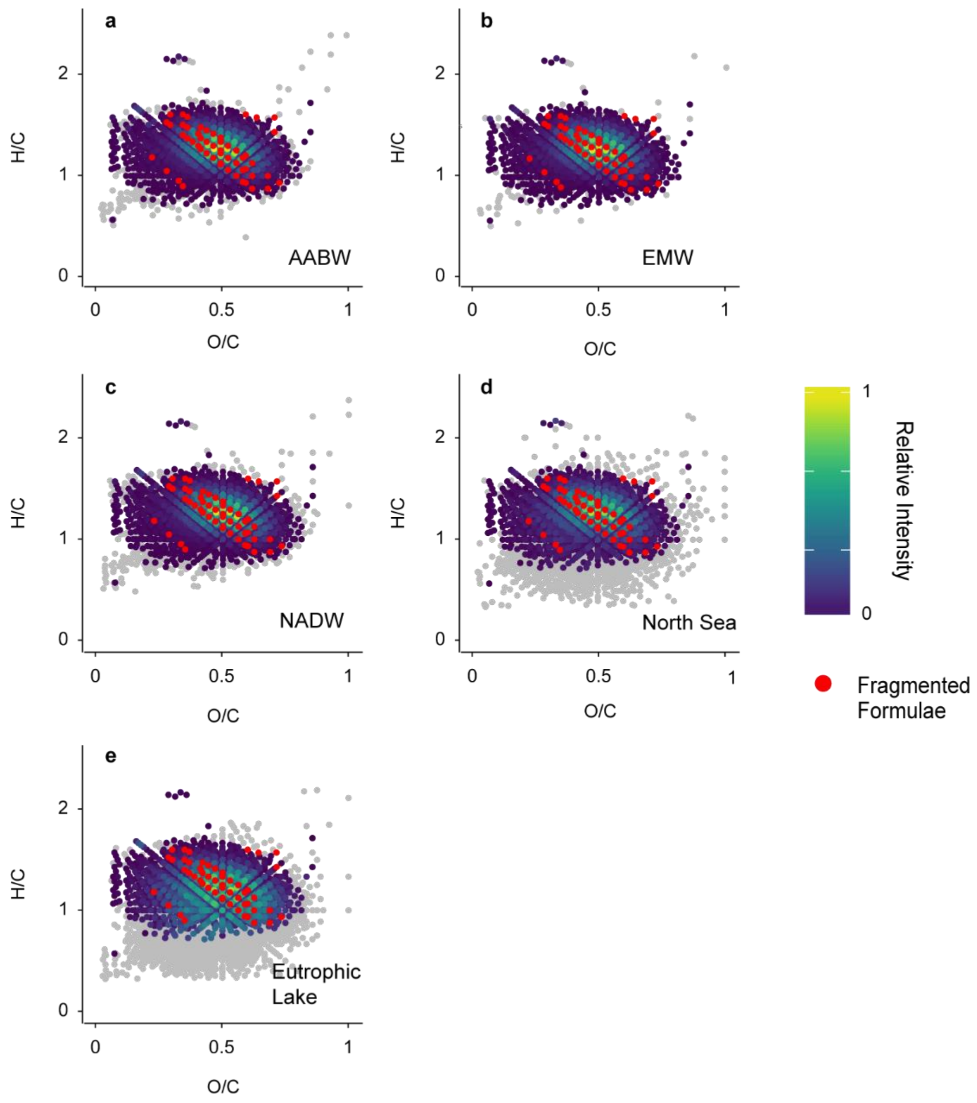

Supplementary Fig. 4: Molecular formula composition of DOM from different environments displayed in van Krevelen diagrams. Each dot represents one (unfragmented) molecular formula in H/C and O/C element ratio space. Color indicates relative signal intensities of the common molecular formulae detected in full range mass spectra (n = 2,531). Grey dots represent compounds that are not occurring in all samples. Red dots mark the molecular formulae that were selected for fragmentation experiments (n = 54). (a) AABW, (b) EMW, (c) NADW, (d) North Sea, and (e) Lake

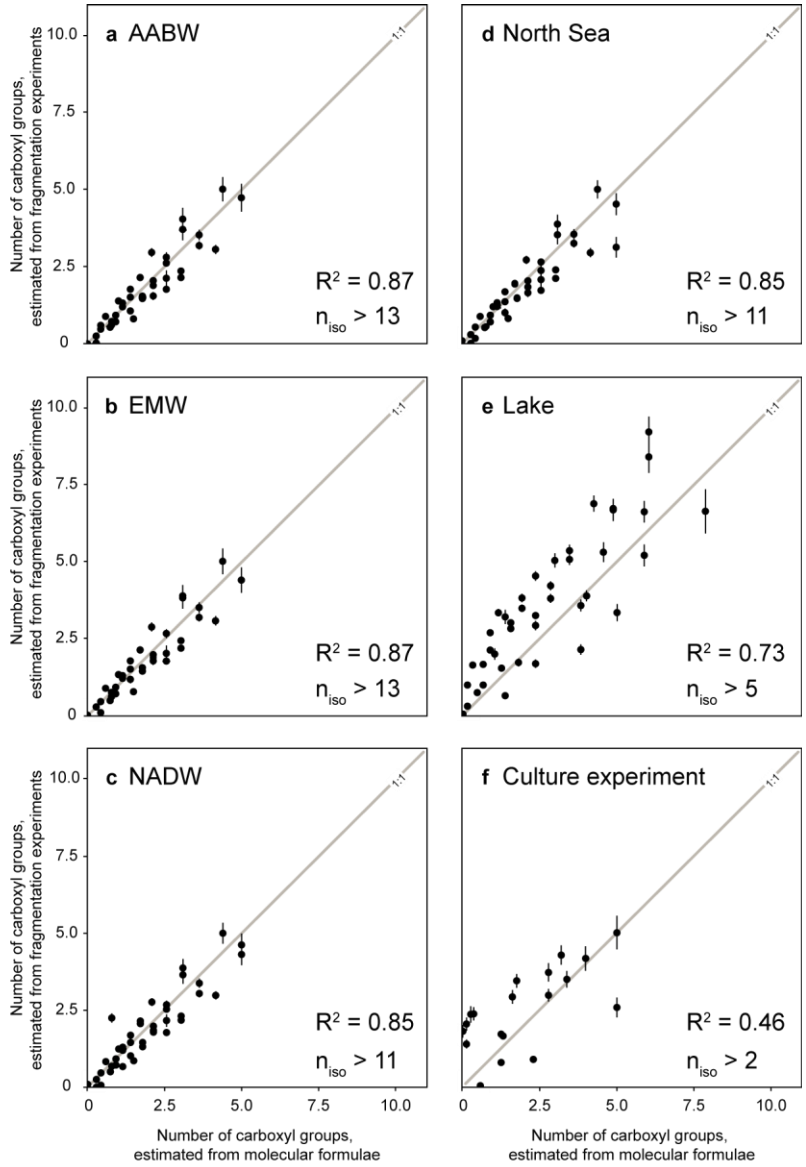

Supplementary Fig. 5: Estimated number of carboxyl groups in DOM compounds from (a) AABW, (b) EMW, (c) NADW, (d) North Sea, (e) Lake, and (f) three-year-long culture experiments[25] estimated from FT-ICR-MS fragmentation data and molecular formulae alone. Each dot in the plot represents one molecular formula (n = 54). Error bars represent analytical variability. The higher the correlation coefficient (R^2) between the two independent estimates, the higher the number of isomers (n_{Iso}) behind a given molecular formula. See Zark et al. (2017)[5] for more detailed description. Osterholz et al.[25] kindly provided data for panel f. Error bars were calculated based on noise plus 5% of the respective fragment ion intensity and under consideration of error propagation.

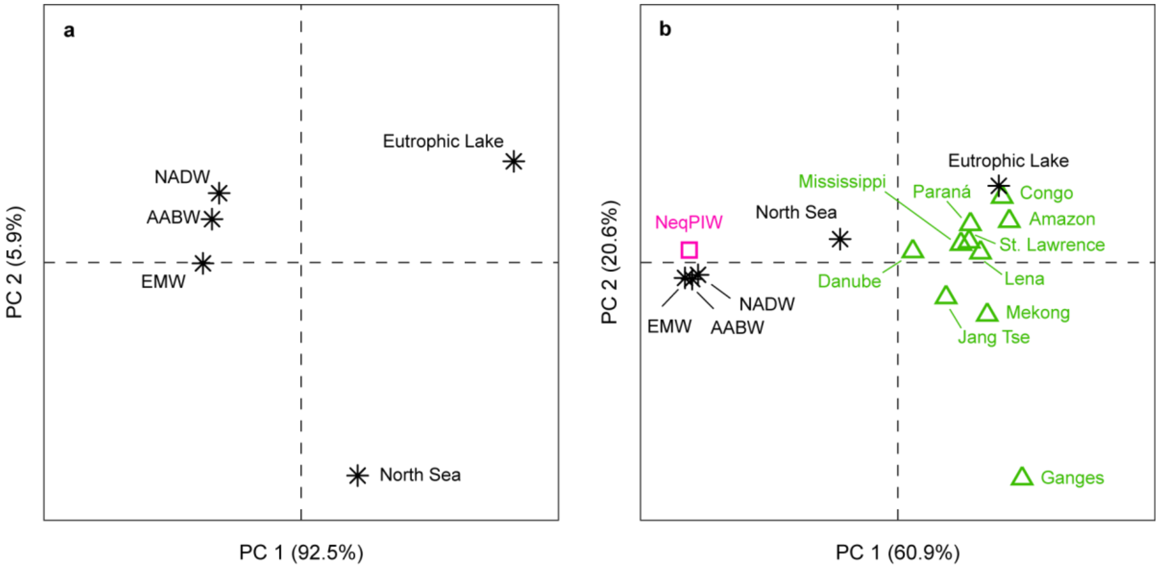

Supplementary Fig. 6: Multivariate analyses of molecular DOM composition using Principal Component Analysis (PCA). Calculations are based on relative signal intensities of all detected molecular formulae in (a) the five samples from different aquatic environments analyzed during our study alone, and (b) together with deep-sea water (NEqPIW) and samples from 10 of the major world rivers (see Riedel et al.[40] for further information). In a) 92% of the total variability of the complex molecular information were summarized in a single component (PC1). This component reflected the different origins of the samples as it correlated significantly with salinity (Pearson, r= 0.91, $p<0.05$, n=5) and DOC concentration of the samples (r=0.90, $p<0.05$, n=5). The results shown in b) reflect that the samples presented in this study span almost the entire range of variability between marine and terrestrial DOM reference samples and are therefore considered representative. NEqPIW = North Equatorial Pacific Intermediate Water.

Supplementary Table 1.

Physical and chemical properties of water samples from different sampling sites.

Sample*	Longitude	Latitude	Depth [m]	SPE-DOC† [µM]	Salinity	# of detected formulae	Common formulae‡ [%]	Aromatic structures§ [%]
AABW	38° 60' W	30° 60' S	4100	23	34.7	3453	73	2.9
	39° 0' W	25° 60' S	4000					
EMW	12° 54' W	35° 14' N	1000	25	36.0	2897	87	2.6
	12° 37' W	38° 45' N	1000					
NADW	22° 46' W	9° 24' N	4000	26	34.9	3932	64	3.3
	22° 46' W	9° 24' N	3000					
	22° 46' W	9° 24' N	2500					
North Sea	7° 54' E	54° 11' N	0	118	32.7	4863	52	9.8
Lake	8° 1' E	53° 12' N	0	1906	0	5396	47	23.8

* All values are for samples pooled at equal parts from the respective positions and depths.

† SPE-DOC is the concentration of the solid-phase extracted DOM fraction in the original water sample.

‡ Common formulae column refers to the 2,531 formulae co-occurring in all samples as percentage of total formulae in a sample.

§ Defined as molecular formulae with an aromaticity index (AI_{mod}) > 0.5. Averages, weighted by corresponding FT-ICR-MS signal intensities, are shown.

Abbreviations: AABW: Antarctic Bottom Water, EMW: Eurafrican Mediterranean Water, NADW: North Atlantic Deep Water, North Sea: Coastal North Sea off Germany, Lake: Zwischenahner Meer, a eutrophic peat lake in North Germany.

Supplementary Table 2.

Molecular characteristics of DOM derived from detected molecular formulae.

Sample	Mean mass* [m/z]	Black Carbon* [%]	Polyphenols O-rich* [%]	Polyphenols O-poor* [%]	Highly unsaturated* [%]	Aliphatic* [%]
AABW	404	0.07	0.27	2.60	91.07	4.94
EMW	404	0.04	0.23	2.35	91.54	5.05
NADW	402	0.07	0.35	2.87	90.75	4.82
North Sea	383	1.32	1.90	6.58	81.69	5.77
Lake	383	4.61	5.99	13.23	70.39	2.74

*Given are means weighted by corresponding signal intensities. Criteria for identifying molecular groups: black carbon AImod≥0.666, no N, S, P; polyphenols O-rich 0.666>AI_{mod}<0.5, O/C>0.5; polyphenols O-poor 0.666>AI_{mod}<0.5, O/C≤0.5; highly unsaturated AI_{mod}<0.5, H/C<1.5, O/C<0.9; aliphatic compounds 2.0≥H/C≥1.5, O/C<0.9, no N. Note that the assignment of molecular formulae to molecular groups is not unambiguous, but gives a helpful overview of likely structures. Other minor categories that are not included in this table are black carbon containing N, S or P, and peptide-molecular formulae (unsaturated aliphatics that contain O and N).
